# Genome‐Wide CRISPR/Cas9 Screening Identifies the COMMANDER Recycling Complex as a Key Player in EV Uptake

**DOI:** 10.1002/jev2.70166

**Published:** 2025-09-23

**Authors:** Miguel Palma‐Cobo, Victor Toribio, Joaquín Morales, Soraya López‐Martín, Carlos Enrich, Albert Lu, María Yáñez‐Mó

**Affiliations:** ^1^ Centro de Biología Molecular Severo Ochoa, IIS‐IP Universidad Autónoma de Madrid, IUBM Madrid Spain; ^2^ Departament de Biomedicina, Unitat de Biologia Cel·lular, Facultat de Medicina i Ciències de la Salut, Centre de Recerca Biomèdica CELLEX, Institut d'Investigacions Biomèdiques August Pi i Sunyer Universitat de Barcelona Barcelona Spain

**Keywords:** cargo delivery, COMMANDER complex, EV uptake, genome‐wide screen

## Abstract

Extracellular vesicles (EVs) hold immense potential in therapeutic delivery, warranting a comprehensive investigation of the mechanisms that regulate their uptake by target cells. To identify key molecular regulators of EV internalization, we conducted a genome‐wide CRISPR (GWC) screen aimed to pinpoint candidate genes that influence EV uptake. We employed a GWC library spanning the entire human genome in K562 cells. 3.6 × 10^12^ EVs isolated from the SKMEL147 human melanoma cell line were labelled with Alexa633‐C5‐Maleimide and incubated for 2 h with 500 × 10⁶ K562 cells, providing a 2000× coverage of the library. The top 5% of high and low fluorescence populations were sorted. Next‐generation sequencing (NGS) was performed to quantify sgRNA enrichment in the sorted populations compared to the unsorted control. Remarkably, among other genes, several members of the COMMANDER complex emerged as significant hits in our screen. We validated the hits in knockout (KO) cell lines of both K562 and HeLa cells using EVs derived either from melanoma or breast cancer cell lines. Kinetic follow‐up of EV cargo, including surface or luminal proteins, suggests that the COMMANDER complex plays a pivotal role in the early stages of EV uptake but also in the final fate of EV components in the target cell.

## 1 Introduction

The cellular secretome, particularly extracellular vesicles (EVs), serves as a major intercellular communication mechanism, exerting profound influence on tissue homeostasis in both physiological and pathological contexts within organisms. These entities wield the capacity to induce a wide range of biological changes on target cells in various dimensions, including metabolism, epigenetics, differentiation, polarity, and motility (Yáñez‐Mó et al. 2015). For effective interplay, EVs may engage with specific signalling receptors on the plasma membrane of the recipient cell, thereby initiating signalling cascades. Alternatively, they may be internalized to exert their effects at intracellular levels. The current state of knowledge underscores that EV uptake represents an active process, with several well‐described pathways, including phagocytosis, micropinocytosis, clathrin‐dependent endocytosis, caveolin‐dependent endocytosis, and lectin‐dependent endocytosis (Mulcahy et al. 2014). The mechanisms involved in EV uptake are contingent upon the state of the recipient cell, encompassing factors such as metabolism, differentiation, and division. In addition, the composition and origin of EVs play a pivotal role, with different uptake pathways potentially acting concomitantly within the same cell. This dynamic interplay underscores the complexity and context‐dependent nature of EV‐mediated intercellular communication.

Previous studies suggest that certain cellular types exhibit high promiscuity in EV uptake, particularly professional phagocytic cells such as macrophages and neutrophils (Di Rocco et al. 2016). These cells predominantly recognize EVs through scavenger receptors, binding to elements like phosphatidylserine—an abundant phospholipid on the outer hemimembrane of most EVs. In contrast, in other cellular types whose physiological functions are less reliant on phagocytosis, the specificity in EV uptake concerning their origin may be higher. For instance, in cancer, research indicates that the presence of various integrins within EVs serves as an indicator of their organotropism. Consequently, EVs containing αvβ5 integrins are selectively taken up by liver cells, while those harbouring α6β1 and α6β4 integrins are preferentially internalized by lung cells (Hoshino et al. 2015, Clares‐Pedrero et al. 2024). All this previous evidence highlights the remarkable diversity of events by which EVs can enter cells. Yet, the molecules that dictate uptake selectivity and specificity remain largely uncharacterized.

Once endocytosed, EVs face three potential fates: the degradative pathway, the secretory pathway via re‐exocytosis, or an ‘escape’ pathway, enabling the vesicular cargo to exert its function in the recipient cell (Clares‐Pedrero et al. 2024). Despite functional experimental observations of these outcomes (Toribio et al. 2019, Bonsergent et al. 2021, Toribio and Yáñez‐Mó 2023), the molecular mechanisms and factors governing the fate decision of endocytosed EVs remain largely unknown. To facilitate the actual transfer of vesicular cargoes, various hypotheses have been proposed. One suggests that vesicles may fuse with the endosome membrane in the acidic environment of late endosomes (Bonsergent et al. 2021), leading to the release of luminal cargo into the cytoplasm (Joshi et al. 2020, Bonsergent et al. 2021). Meanwhile, membrane cargo would remain on the endosomal membrane, potentially recycled to the plasma membrane or to other organelles. Another possibility involves a specific mechanism directing EVs to various organelles, such as the endoplasmic reticulum, Golgi, or nucleus. Notably, recent discoveries highlight a mechanism involving Rab7 (a late endosome‐specific GTPase), VAP‐A (a ER‐resident protein involved in endosomal and lipid trafficking), and ORP‐3 (an intracellular cholesterol sensor), which collaborate to transport endocytosed vesicles to the nucleoplasmic reticulum (Santos et al. 2019, Rappa et al. 2017). This opens the door to the possibility of similar mechanisms redirecting vesicles to other subcellular locations.

Despite considerable progress in this field, there remains a lack of comprehensive characterization regarding how EV uptake functions and the variables influencing this process. Identifying new molecular candidates involved in each phase of the process is essential. Previously, we pioneered the use of genome‐wide CRISPR/Cas9 screening (Lu et al. 2018) to systematically and unbiasedly investigate factors involved in EV biogenesis and release. In this study, we extend this approach to explore EV uptake on an unprecedented genome‐wide scale. While pooled CRISPR screening and fluorophore‐based EV labelling are well‐established techniques in their respective fields, their application to studying EV uptake is novel and represents a significant advancement in EV biology. To the best of our knowledge, no prior study has employed a genome‐wide CRISPR screening approach to systematically identify cellular factors involved in EV uptake. Most studies in this field have relied on lower‐throughput, hypothesis‐driven approaches, which, while valuable, lack the unbiased, large‐scale discovery potential of pooled CRISPR screens. With this approach, we unveiled an unexpected role of the COMMANDER complex and other intracellular regulators in EV uptake and cargo delivery. This work thus provides an exciting starting point to monitor EV uptake in a systematic and unbiased fashion, thereby advancing our understanding of this intricate cellular process.

## 2 Materials and Methods

### 2.1 Antibodies and Staining Reagents

Primary antibodies used were mouse hybridomas‐conditioned media anti‐CD9 (VJ1/20) and anti‐CD63 (Tea3/10) (Suárez et al. 2017), anti‐CD151 (VJ1/16), anti‐β1 integrin (TS2/16), anti‐α1 integrin (TS2/7), anti‐ α2 (Tea 1/41.1), anti‐α3 integrin (VJ1/6.1), anti‐α5 integrin (P1D6), anti‐ICAM (MEM111), anti‐ALCAM (P15) (Suárez et al. 2017, Domínguez et al. 2010) and anti‐CD81 (5A6) (provided by Dr S Levy, Stanford, USA). Other primary antibodies used were anti‐ARF‐6 (mouse monoclonal, Sigma, A5230), anti‐VDAC (Rabbit polyclonal, Abcam, ab154856), anti‐Calnexin (Rabbit polyclonal, Enzo, ADI‐SPA‐865‐F), anti‐LAMP2 (Rabbit polyclonal, Abcam, ab18528), anti‐COMMD2 (Rabbit, Sigma‐Aldrich, HPA044190), anti‐COMMD3 (Rabbit, Bioss Antibodies, bs‐8183R), anti‐COMMD5 (Rabbit polyclonal, Invitrogen, PA1‐46178/Rabbit, Proteintech, 10393‐1‐AP), anti‐COMMD8 (Rabbit, Invitrogen, PA5‐57446), anti‐COMMD10 (Rabbit, Abcam, ab127691/Rabbit, GeneTex, GTX121488), anti‐CCDC22 (Rabbit, Abcam, ab224038), anti‐CCDC93 (Mouse, Santa Cruz, sc‐514600/Rabbit, Preoteintech, 20861‐1‐AP), anti‐VPS35L (Rabbit, Abcam, ab97889), anti‐WDR91 (Rabbit, Abcam, ab80614), anti‐AMBRA1 (Mouse, Santa Cruz, sc‐514600), anti‐EEA1 (Mouse, BD Biosciences, 610456). Conjugated primary antibodies anti‐CD9 (VJ1/20, Immunostep, 9PE‐100T, 0.5 µg) and anti‐CD81 (MEM‐38, Immunostep, 81PE‐100T, 0.5 µg). As secondary antibodies the following were employed: GαMouse HRP and GαRabbit HRP (Thermo Scientific) (1/5000 for immunoblotting), GαM Alexa 488 (Thermo Fisher, A‐21121, 1/200), DαR Alexa 555 (Thermo Fisher, A‐31572, 1/200), DαR Alexa 488 (Thermo Fisher, A‐21206, 1/200), DαM Alexa 647 (Thermo Fisher, A‐31571, 1/200), Phalloidin Alexa 350 (Thermo Fisher, A22281, 1/200) and LysoTrackerTM Green DND‐26 (Thermo Fisher, L7526).

### 2.2 Cell Culture and Media

SKMEL‐147 melanoma cell line was cultured in DMEM supplemented with 10% FBS and 1% penicillin/streptomycin. K562 leukaemia cell line was cultured in RPMI supplemented with 10% FBS, 1% penicillin/streptomycin and 2 mM L‐glutamine. HeLa cell line was cultured in DMEM supplemented with 10% FBS, 2 mM glutamine and 1% penicillin/streptomycin. Breast cancer cell line SUM159 was cultured in DMEM F‐12 culture medium supplemented with 5% FBS, 1% penicillin/streptomycin, non‐essential amino acids (80 mg/mL) (HyClone GE Healthcare), HEPES (10 mM), insulin (5 µg/mL) and hydrocortisone (1 µg/mL). For EV isolation from conditioned media or EV uptake assays, their corresponding culture medium was supplemented with EV‐depleted FBS, obtained by ultracentrifugation at 100,000 × *g* ON at 4°C and filtration with a 0.22 µm pore diameter filter (SLGS033SS, Millex).

### 2.3 EV Isolation

SKMEL‐147 or SUM159 cells were cultured in p150 culture plates with 20 mL of the corresponding medium supplemented with EV‐depleted FBS and left in culture for 6 days. Cells were seeded at a low density to reach confluence on the day of collection of the conditioned media. Cell viability was above 95% in all experiments. Conditioned media were precleared by centrifugation at 200 × *g* for 5 min at 4°C to remove cells and at 3200 × *g* for 30 min at 4°C to eliminate cellular debris before EV isolation by ultracentrifugation or size exclusion chromatography:

#### 2.3.1 Ultracentrifugation (UC)

Total EVs were isolated from precleared conditioned media by ultracentrifugation at 100,000 × *g* for 2 h in a Beckman L8‐70 M ultracentrifuge using an SW‐Bucket AH‐627 rotor. For differential isolation of large and small EVs from SUM159 conditioned media, an intermediate step of ultracentrifugation at 10,000 × *g* for 1 h was performed. Pellets were resuspended in 0.22 µm filtered PBS.

#### 2.3.2 Size Exclusion Chromatography (SEC)

Precleared conditioned media were concentrated by ultrafiltration at 2000 × *g* for 30 min using Amicon Ultra‐15 filters (100K, Millipore, Billerica, MA, USA) to a final volume of 250 µL. The concentrated supernatant was then loaded onto a 1 mL size exclusion chromatography column, using 4% Rapid Run Agarose Bead Fine (Agarose Bead Technologies, 4RRF‐X). Twelve fractions of around 100 µL were collected, and EV‐positive fractions were detected by dot blot analysis with EV‐specific markers (CD81, CD9 or CD63).

### 2.4 EV Staining

EVs were labelled using Alexa 633 C5‐Maleimide reactive (25 µM) (Thermo Fisher, A20342), using an EV concentration of 3.7 mg/mL, ON at 4°C. Dye remnants were removed by passing the sample through an Exo Spin column with an exclusion pore of 3000 MW (Invitrogen) or by SEC. EVs’ intraluminal cargo was stained using either commercial probes, SBI's ExoGlow‐Protein EV Labelling Kit or carboxyfluorescein succinimidyl ester (CFSE) (Life Technologies, C34554) at 5 mM for 1 h at room temperature.

### 2.5 EV Analysis

#### 2.5.1 Nanoparticle Tracking Analysis (NTA)

EVs concentration and size were assessed using a NANOSIGHT LM10 (Malvern Instruments Ltd, Malvern, UK) equipped with a camera charge‐coupled device (CCD, model F‐033) and a 638 nm laser. NTA 3.0 software was used for the analysis. Three videos of 60 s each were recorded with the camera level set at 12 and analyzed with a detection threshold of 10.

#### 2.5.2 Transmission Electron Microscopy (TEM)

EV samples obtained by SEC from SKMEL‐147 conditioned media were adsorbed on carbon‐coated nickel grids by floating an ionized grid onto a drop of the sample. The grids were contrasted with 2% uranyl acetate. Samples were visualized in a Jeol JEM‐1010 (Jeol, Japan) at 80 Kv, and images were acquired with a 4KI 4K CMOS camera, F416, from TVIPS (Gauting, Germany).

#### 2.5.3 Western Blot

Cells or EV's ultracentrifuged pellets were lysed with TBS + 1% Triton X‐100 containing protease inhibitors (Roche) at 4°C for 20 min. Protein concentration of lysates was determined using Pierce BCA Protein Assay kit (Thermo Scientific). Lysates were boiled in non‐reducing Laemmli buffer at 96°C for 5 min, and 20 µg of lysates were loaded in a 10% polyacrylamide SDS‐PAGE gel. After electrotransference, PVDF membranes were blocked with 5% skimmed milk diluted in TBS and 0.1% Tween‐20 for 20 min. Correspondent primary antibodies were incubated ON at 4°C. Secondary antibodies diluted in 5% skimmed milk in TBS, 0.1% Tween‐20 were incubated at 30’ RT. Immunoblots were revealed with Super Signal West Femto HRP substrate (Thermo Scientific), and images were acquired with a LAS 4000 mini system (General Electric).

#### 2.5.4 Single Vesicle Flow Cytometry

SKMEL‐147 EVs isolated by SEC or filtered PBS (procedural control) were stained with Alexa 633 C5‐Maleimide as described above. Samples were then immunostained with anti‐CD9‐PE or anti‐CD81‐PE antibodies and passed through an SEC column in order to get rid of non‐bound antibodies or antibody clumps. BD FACSymphony A1 flow cytometer was used to analyse the samples.

#### 2.5.5 EV Immunofluorescence

2.5 µg or 1.8 × 10^9^ of A633 C5‐Maleimide stained EVs in 250 µL were spun onto coverslips using a CytoSpin at 2000 rpm for 5 min and immediately fixed with 4% PFA in PBS for 10 min, blocked for 10 min with PBS and 1% BSA, and incubated with appropriate primary antibodies (1 h at RT), and secondary antibodies were incubated (30 min at RT in dark).

### 2.6 EV Uptake Assays

#### 2.6.1 Confocal

For EV uptake visualization by confocal microscopy, 2.5 × 10^5^ K562 cells/well were seeded in a p24 plate with RPMI supplemented with EV‐depleted FBS at a concentration of 10^6^ cells/mL and then incubated with 2 µg of A633 C5‐Maleimide‐stained EVs for 2 h at 37°C. At this time point, cells were centrifuged, washed with PBS and treated with 0.05% trypsin/0.016% EDTA for 2 min at RT to eliminate EVs attached to the cell surface, and resuspended in PBS at a concentration of 40 cells/µL. 10^4^ cells were seeded per coverslip by using a CytoSpin (SHANDON) at 800 rpm for 5 min. Samples were immediately fixed with 4% PFA in PBS for 10 min, blocked with PBS, 1% BSA, and 0.1% saponin, and stained with primary and secondary antibodies. Images were acquired with an LSM880 (Zeiss) inverted confocal microscope and a Plan‐Apochromat 63x/1.4 Oil DIC M27 objective.

For live cell time‐lapse microscopy, HeLa cells (EMPTY or COMMD5KO) were transfected with CD63‐Cherry PCDH‐CMV plasmid using jetPEI (Polyplus). Melanoma‐derived EVs were stained as previously described with both Alexa 633 C5‐Maleimide and CFSE. The day before the experiment, 1.5 × 10^5^ HeLa cells were seeded in a μ‐Slide 8 Well (ibidiTreat, 80806). Medium was replaced by EV‐depleted medium without phenol red before the addition of the labelled EVs (20,000 EVs/cell). Imaging was performed using an Olympus SpinSR10 microscope with a SoRa disc and a 100X/1.45 Oil Extended‐Apo (UplanXApoO) objective at 37°C and 5% CO_2_. A stack of three confocal planes distanced 0.5 µm in the *z*‐axis from 5 cells from each condition was acquired every minute.

Tracking and statistical analysis of the confocal videomicroscopy uptake assays were performed using ImageJ 1.54p with Java 1.8.0_322. Maximal intensity Z projections were used, and a substack of the frames 60–160 min was analyzed. Cell segmentation was performed using the CD63 channel with the MinError thresholding algorithm to create a mask and cell area. The cell mask was applied to the maleimide‐C5 AF633 and CFSE channels to track those EVs in contact with transfected cells. Thresholds of noise fluorescence were applied, and to follow the double positive structures, the values of both channels were multiplied. Structures bigger than 2 pixels were tracked using TrackMate 7.14.0 plugin. Trackmate detection algorithm was LoG. LAP tracker was used, with a frame‐to‐frame max distance of 4 µm, gap closing distance of 6 µm and max frame gap of 2.

#### 2.6.2 Flow Cytometry

K562 sgRNAs CRISPR cell library development and establishment have been previously described in (Lu et al. 2021). This library encompasses 10 different sgRNAs for each gene in the human genome, alongside various negative controls (non‐targeting and safe‐targeting controls). The total library is thus composed of around 211,000 single sgRNA elements. Parental K562 cell line stably expresses SpCas9. Two independent flow cytometry EV‐uptake assays were performed with 500 × 10^6^ cells from the cell library, at a concentration of 10^6^ cells/mL in RPMI supplemented with 10% EV‐depleted FBS and incubated with 4.8 mg (around 3.6 × 10^12^ EVs) of Alexa 633 C5‐Maleimide‐stained EVs for 2 h at 37°C. After the incubation time, cells were centrifuged, washed with PBS and treated with trypsin for 2 min at RT to eliminate EVs attached to the cell surface. Finally, cells were resuspended in sorting buffer (PBS with 0.1% BSA). FACSAria and FACSsorp flow cytometers were used. Both the 5% populations with the lower and the higher A633 fluorescence signals from the total population were sorted. After sorting, samples were pelleted and frozen at −80°C along with a non‐sorted population and a complete cell library control.

For hit validations, 250,000 KO K562 or HeLa cells and 5 × 10^9^ maleimide‐stained melanoma or breast cancer EVs were employed per condition.

For the kinetic flow cytometry uptake assay, both surface (Alexa 633 C5‐Maleimide) and intraluminal EV cargo (ExoGlow‐Protein EV Labelling Kit) melanoma EVs were separately stained as previously described. HeLa cells were seeded the previous day (4 × 10^4^ cells/well in a p24 plate). Then, cells were fed with the stained EVs, upon changing the media to EV‐depleted FBS‐containing media at a ratio of 1 × 10^4^–2 × 10^4^ EV/cell at each time point. Cells were then detached and washed in PBS at 4°C. Flow cytometry analysis was performed using a spectral cytometer, Aurora 4L (Cytek Biosciences, Fremont, CA, USA).

#### 2.6.3 Bioluminescence

2.5 × 10^5^ K562 or HeLa cells were resuspended in 300 µL of EV‐depleted medium with 60 µM of EnduRen (Promega, E6482) and left 2 h at 37°C in the incubator for reagent internalization and chemical deprotection by intracellular esterases. Then, cells were washed and resuspended in 300 µL of EV‐depleted medium and split into two wells (1.25 × 10^5^ cells/well) of a white tissue culture‐treated p96 well plate with clear bottom (Corning Inc., 3610). Then 2.3 × 10^9^ SKMEL‐147 EVs carrying mEmerald‐CD9‐Rluc construct or SUM159 EVs carrying either CD9DSP1+DSP2 or CD63DSP1+DSP2 (Toribio and Yáñez‐Mó 2023) were added to each well. A control of EVs incubated with EnduRen was also included. Samples were incubated for 4 h at 37°C, and the luminescence signal was measured with a Tecan GENios Microplate reader, with 5000 ms of exposure time and a gain of 150.

### 2.7 Next Generation Sequencing and Analysis

gDNA of all six populations was isolated. For unsorted populations (Unsorted 1, Unsorted 2) that contained 60 × 10^6^ cells, QIAamp DNA Blood Maxi Kit (Qiagen Cat# 51192) was used. For FACS‐sorted populations (Low 1, Low 2, High 1 and High 2), which were all around 10 × 10^6^ cells, QIAamp DNA Blood Mini Kit (Qiagen Cat# 51104) was employed.

sgRNAs integrated into genomic DNA (gDNA) of the different populations were amplified by a first large‐scale PCR using primers targeting flanking regions (U6 promoter and tracrRNA sequence), in which the entire isolated gDNA of each sample was used in multiple 100 µL PCR reactions (find primer sequences in Table S1). For the unsorted gDNA samples, 40 reactions were set up per screen replicate (*n* = 2). For the gDNA samples from the sorted populations (Low 1, Low 2, High 1 and High 2), around 5–15 reactions were set up per sample, depending on the gDNA recovery. For each reaction: dNTPs (100 µM), gDNA (10 µg), 5x Herculase buffer, Herculase II Fusion DNA Polymerase (Agilent, 600677), Forward primer _oMCB1562 (1 µM), Reverse primer_ oMCB1563 (1 µM). (98°C/2 min) × 1, (98°C/30 s, 59.1°C/30 s, 72°C/45 s) × 18 cycles, (72°C/3 min) × 1. All the PCR amplicons of their respective samples were pooled and mixed. The PCR product, a band around 300 bp, was checked by electrophoresis in a 2% agarose gel. A second PCR was performed to further amplify the sgRNAs and to add the sequencing overhangs. Only one 100 µL reaction for each sample was prepared, using 5 µL of the mixed amplicon obtained in the previous PCR along with dNTPs (500 µM), 5x Herculase buffer, Herculase II Fusion DNA Polymerase, Forward primer_oMCB1439 (800 nM), Reverse barcoded primer (800 nM). (98°C/2 min) × 1, (98°C/30s, 59.1°C/30s, 72°C/45s) × 20 cycles, (72°C/3 min) × 1. Half the PCR products were loaded and run in a 2% agarose gel electrophoresis to check the 275 bp band of the PCR product, which was excised, and amplicons purified using MinElute PCR Purification Kit (Qiagen, Cat#28004).

An aliquot of the amplified sgRNAs from each sample was quality checked on a Bioanalyzer 2100 (Agilent), titrated by qPCR and pooled together in equimolecular amounts for their sequencing on a NovaSeq 6000 (Illumina) using a NovaSeq 6000 SP Reagent Kit v1.5 (100 cycles) (Illumina, 20028401) and a PAGE‐purified custom sequencing primer.

The resulting FASTA sequences from the different samples were mapped to the sgRNA library sequence list using Bowtie; the identified sequences were counted with Corset, and then the differential expression analysis was done using EdgeR (Empirical Analysis of Digital Gene Expression Data in R).

### 2.8 Knock‐Out Cell Lines Generation

Knock‐out cell lines were generated using lentiviral vector TLCV2 (Addgene #87360) with the corresponding sgRNA for each gene: AMBRA1 (Exon 4) (5’ GCCAATGAGATCCACTTC 3’), COMMD2 (Exon 4) (5’ GTACATCTAGTCGCCATTCA 3’), COMMD3 (Exon 5) (5’ GATCTCTCCCTCATATAA 3’), COMMD5 (Exon 1) (5’ GTGTGTGCATGCCTGCCAGC 3’), COMMD8 (Exon 3) (5’ GGAAAGATGAAATCAAAC 3’), COMMD10 (Exon 7) (5’ GAGACTATACAAGCACAGC 3’), CCDC22 (Exon 2) (5’ GTCTGCCCGGTTCCGCC 3’), CCDC93 (Exon 3) (5’ GTAGTAGGAGGAATGACT 3’), VPS35L (Exon 4) (5’ GGATTTCTCCCCGTTTGT 3’), WDR91 (Exon 2) (5’ GTTAATGCAGGTGTATGACT 3’). Insertion of the sgRNA in TLCV2 plasmid was performed following the LentiCRISPRv2 protocol (https://media.addgene.org/cms/filer_public/53/09/53091cde‐b1ee‐47ee‐97cf‐9b3b05d290f2/lenticrisprv2‐and‐lentiguide‐oligo‐cloning‐protocol.pdf).

For lentivirus production, around 8 × 10^6^ of HEK‐293T cells were seeded in a p150 plate and cotransfected on the next day with the plasmids pMD2.G (Addgene #12259), pCMV delta R8.2 (Addgene #12263) and TLCV2 containing the corresponding sgRNAs, diluted in 500 µL of NaCl (150 mM) and mixed with 60 µL of jetPEI (Polyplus), also diluted in 500 µL of NaCl (150 mM). 48 h post‐transfection cell supernatant was collected, filtered through 0.45 µm filters (Acrodisc, PALL) and stored at 4°C. 15 mL of fresh media was added to the cells to recollect conditioned media 72 h post‐transfection. Viral particles were concentrated by 2 h of ultracentrifugation at 4°C and 93,000 × *g* with an AH‐627 rotor, resuspended in cold PBS and stored at −80°C.

Cells to be transduced were seeded on a p6 multiwell plate in their corresponding culture medium with 8 µg/mL polybrene (Santa Cruz) and the lentivirus at a multiplicity of infection (MOI) between 0.8 and 1.5. Plates were centrifuged at 32°C and 890 × *g* for 70 min and placed into the incubator at 37°C. Transduced cells were selected by a 5‐day puromycin (1 µg/mL) treatment. The expression of the CRISPR machinery (Cas9 and sgRNA) was induced by treating the cells with 2 µg/mL doxycycline every 2 days during a total of 14 days. To increase the yield of KO population, cells were sorted with FACSAria Fusion cell sorter (BSC II, BD Biosciences, San Jose, CA, USA) to keep those expressing the Cas9‐GFP fusion protein. After expanding the cells post‐sorting, gene deletion was checked by WB.

### 2.9 Cell Flow Cytometry

Cells were trypsinized, washed with PBS and incubated with the primary antibody of interest for 1 h at 4°C. After being washed with PBS, cells were incubated with the appropriate secondary antibodies (ThermoFisher Scientific) for 30 min at 4°C. Cells incubated with only the secondary antibody were used as a negative control.

For lysosomal analysis by flow cytometry, cells were plated 48 h in advance, and for the starving condition, medium was changed to medium without FBS 24 h prior to the staining. Samples were stained with LysoTracker Green (Invitrogen) following the manufacturer's instructions with a concentration of 75 nM in serum‐free culture medium for 10 min at 37°C. Analyses were performed in a FACSCanto A (BD Biosciences).

### 2.10 Confocal Microscopy

Coverslips were coated with 5 µg/mL fibronectin (Sigma‐Aldrich) in p24 well plates for 30 min at 37°C, and 20000 HeLa cells/well were seeded and cultured ON at 37°C, 5% CO_2_. Samples were then washed with PBS and fixed with 2% paraformaldehyde for 10 min at RT. Coverslips were washed with TBS several times, incubated with a blocking solution (5% BSA, 0.1% saponin, in PBS) for 30 min at RT in a wet chamber, washed with PS (15 mM glycine, 0.1% saponin, 10 mM HEPES, 0.5% BSA in PBS) and incubated with the primary antibody diluted in PS ON at 4°C. After washing with PS, the appropriate secondary antibody diluted in PS was added onto the coverslips for 1 h RT. Secondary antibody was washed with PBS before incubation with phalloidin diluted in PBS for 1 h. Coverslips were finally washed with PBS and placed onto slides using ProLong Glass Antifade Mountant (Thermo Fisher, P36984). Imaging was performed in a confocal microscope, Zeiss LSM900, with a 100X objective (Zeiss 420792–9900). Images were acquired with a voxel size of 31.2 × 31.2 × 130 nm (x,y,z) for deconvolution using Huygens Professional (Scientific Volume Imaging, v24.10) to enhance image resolution and contrast. A theoretical point spread function (PSF) was generated based on the microscope optical parameters, and deconvolution was performed using the classical maximum likelihood estimation (CMLE) algorithm with 40 iterations per channel. Background levels were set manually in each channel: phalloidin to 800 units and EEA1 to 2000 units, and the signal‐to‐noise ratio (SNR) to 29.13 and 13, respectively, to preserve structural detail while reducing out‐of‐focus blur.

Endosomal structure analyses were performed in ImageJ 1.54p with Java 1.8.0_322. A maximum intensity Z‐projection of deconvolved stacks was performed. Cell segmentation was performed using the phalloidin channel with the MinError thresholding algorithm to create a mask and cell area. This mask was used on the EEA1 channel to analyze EEA1^+^ structures in the cell, thresholded with the Triangle dark algorithm and subdivided using watershed. Particles bigger than 0.01 µm2 were taken into account for statistical analyses.

## 3 Results

### 3.1 Exploring Molecular Players in Extracellular Vesicle Uptake Through a Genome‐Wide Genetic Screening

To identify the molecular determinants governing EV uptake, we conducted a genome‐wide CRISPR/Cas9 (GWC) screen. While prior research—including our own (Lu et al. 2018)—has utilized high‐throughput methods, including CRISPR/Cas9‐based approaches, to study EV biogenesis and release, high‐throughput screening of EV uptake mechanisms remains largely unexplored. Most studies in this field have relied on lower‐throughput, hypothesis‐driven approaches (De Jong et al. 2020), which, while valuable, lack the unbiased, large‐scale discovery potential of pooled CRISPR screens.

For our screen, total EVs were isolated by ultracentrifugation from conditioned media of the melanoma cell line SKMEL‐147 and characterized according to the MISEV recommendations (Théry et al. 2018, Welsh et al. 2024). They presented an average size of 162 nm, as assessed by NTA (Figure S1A), consistent with their appearance in transmission electron microscopy (Figure S1B). Additionally, purified EV fractions were enriched in canonical markers such as tetraspanins CD63, CD9 and CD81 or ARF6, while lacking common EV‐negative markers calnexin and VDAC (Figure S1C).

**FIGURE 1:**
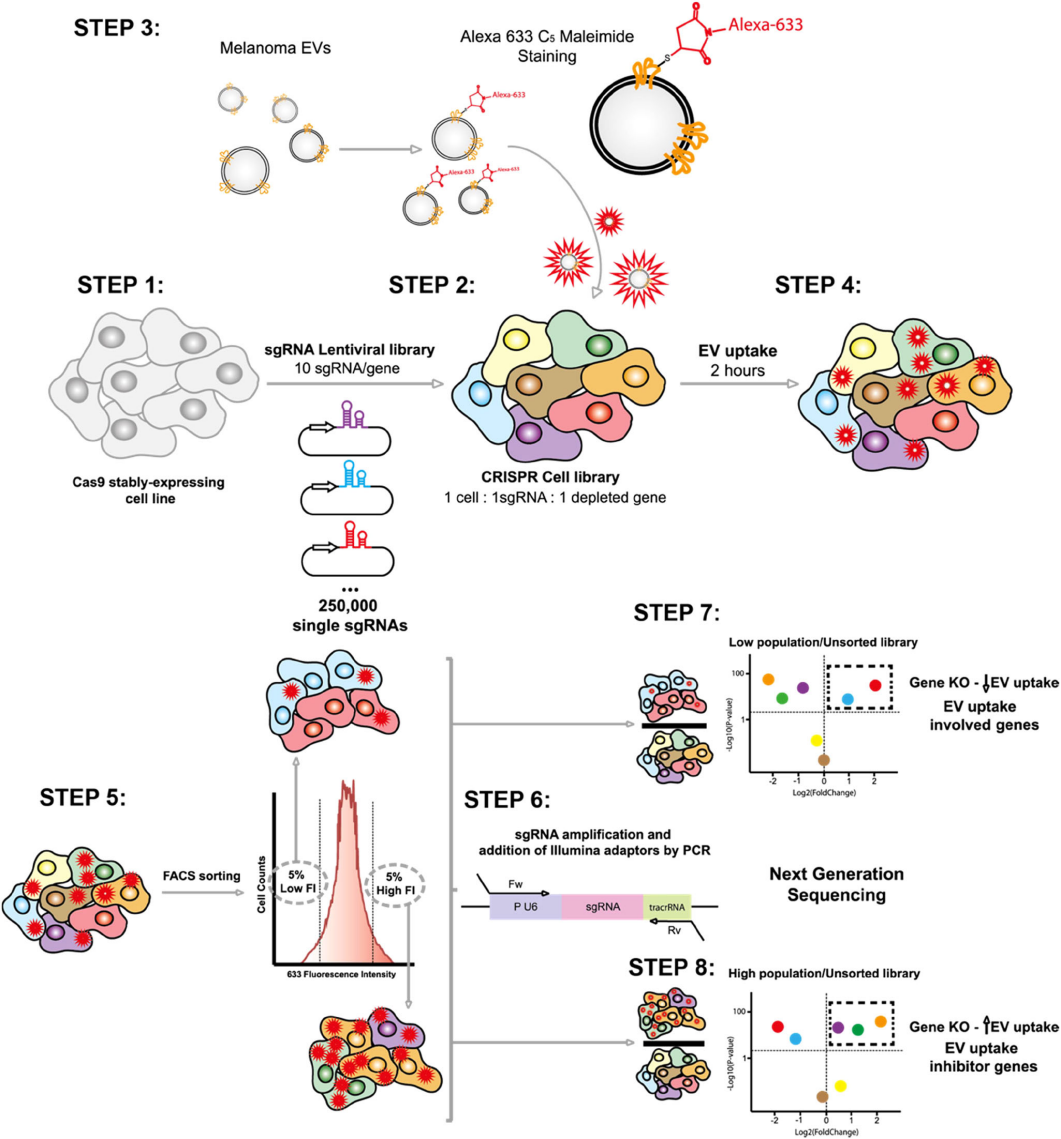
Workflow scheme of the genetic screening of a GWC cell library by a flow cytometric EV uptake assay. **STEP 1**: A K562 cell line stably expressing the endonuclease SpCas9 is transduced with a lentiviral sgRNA library of the CRISPR system, which in this case targets the whole human genome, containing 10 sgRNAs per gene and ∼12000 negative control sgRNAs, to form a library containing about 250,000 elements. **STEP 2**: After transduction, cells were selected with puromycin and by flow cytometry by co‐expression of CFP. Finally, a CRISPR cell library was established, in which each cell had one sgRNA inserted in its genome and as a consequence of the expression of the CRISPR machinery, only one deleted gene. **STEP 3**: EVs isolated from conditioned media from the melanoma cell line SKMEL‐147 are labelled with the compound Alexa 633 C5‐Maleimide, which chemoselectively binds to ‐SH groups. **STEP 4**: The CRISPR cell library was incubated with the fluorescently labelled EVs for 2 h. **STEP 5**: A flow cytometer was used to sort from the total population, the 5% with the highest fluorescence signal (high) and the 5% with the lowest fluorescence signal (low). **STEP 6**: Genomic DNA was extracted from both the sorted populations and the unsorted control library, then the inserted sgRNAs were isolated by PCR and analyzed by Illumina next‐generation sequencing. Genes involved in EV uptake **STEP 7**: or genes that inhibit this process **STEP 8**: were inferred by comparing sgRNA enrichment in both sorted populations (low and high) to that in the unsorted population.

The EV samples were stained with Alexa 633 C5‐Maleimide, a small molecule that under neutral pH conditions (6.5–7.5) selectively binds covalently to protein sulfhydryl residues. This staining was evaluated using single‐vesicle flow cytometry and confocal fluorescence microscopy, demonstrating co‐staining with EV markers CD81, CD9, CD63 and LAMP2 (Figure S2). The covalent maleimide staining facilitated the visualization of EV uptake via confocal microscopy (Figure S3A) and its quantification by flow cytometry (Figure S3B).

To identify potential molecular candidates involved in EV uptake, we utilized a K562 cell line transduced with a genome‐wide CRISPR library as our EV receptor cells. This library encompasses 10 different sgRNAs for each gene in the human genome, alongside various negative controls (non‐targeting and safe‐targeting controls). After transduction, cells were selected with puromycin and monitored by flow cytometry according to CFP co‐expression to establish a CRISPR cell library in which each cell was transduced with a single sgRNA, resulting in the knockout of a single gene (Figure 1).

For robust sequencing coverage, we performed the EV uptake assay with 500 × 10^6^ target cells from the transduced library, providing a 2000x coverage for each sgRNA. Cells were exposed to Alexa 633 C5‐Maleimide‐stained EVs for 2 h, and subsequently, the 5% lower and higher A633 fluorescence populations were sorted by flow cytometry (Figure 1). Approximately 10 × 10^6^ cells were sorted into both low and high fluorescence populations, while the unsorted population consisted of approximately 60 × 10^6^ cells. Two independent experiments were conducted. Illumina sequencing of the unsorted library revealed a coverage of nearly 100% of the original sgRNA library list, confirming that the entire genome was well‐represented in the screening library and that genes associated with growth impairment had not been inadvertently depleted from the original library due to population drift. The low‐fluorescence population likely contained cells that had taken up fewer stained EVs due to the lack of expression of a gene involved in EV uptake, whereas the high fluorescence population represented cells that had taken up more EVs due to the absence of expression of a gene inhibiting EV uptake (Figure 1).

Genomic DNA from the different populations (low, high and unsorted) from the two independent experiments was isolated, and the sgRNAs within the genomic DNA were amplified by PCR. Subsequently, sequences were further amplified in a second PCR step, incorporating sample barcodes and Illumina sequencing overhangs (P7 and P5). Samples (low, high and unsorted) were pooled, and Illumina sequencing was performed using a common custom primer (Figure 1).

### 3.2 The COMMANDER Complex Emerges as a Prominent Hit in the Screen

**FIGURE 2:**
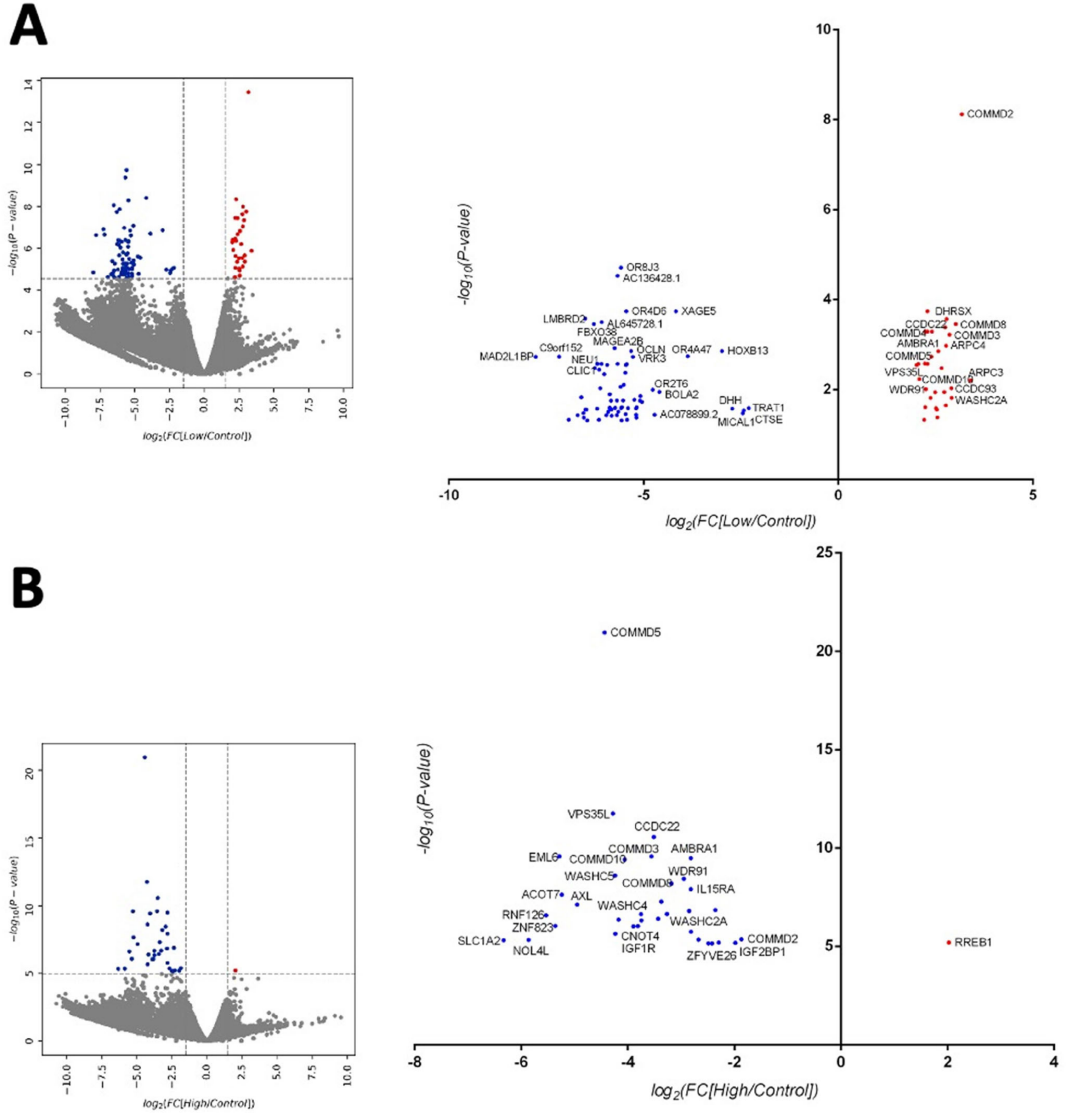
Differential expression analysis of low‐ and high‐fluorescence populations. Low/Control (A) or high/control (B) data sets were obtained after a differential analysis of the count of sgRNAs by NGS present in the low or high fluorescence population (low or high) with respect to the unselected library performed in two independent experiments. Volcano plots of both populations, marking with dashed lines the established thresholds, are shown. On the ordinate axis is marked the value of ‐Log_10_[FDR = 0.05], and on the abscissa axis the values of Log_2_[FC = 0.666] as the lower threshold and Log_2_[FC = 1.5] as the upper threshold. In blue are marked those sgRNAs that are underrepresented in each population, and in red those that are significantly enriched with respect to the control. An enlarged area of the volcano plot on the right shows only those sgRNAs that exceeded the established thresholds.

The sgRNA counts from both the low and high sorted populations were compared with those from the unsorted library, which achieved nearly 100% coverage of the original sgRNA library list. Plotting the data on volcano plots revealed that the low/control and high/control populations exhibited a normal distribution. Only sgRNAs with a fold change (FC) exceeding ±1.5 and a false discovery rate (FDR) below 0.05 were deemed as potentially relevant genes for EV uptake (Figure 2). In the high/control dataset, only one sgRNA showed enrichment compared to the unsorted control, corresponding to RREB1 (Ras Responsive Binding Protein 1) (Figure 2B, right panel).

**FIGURE 3:**
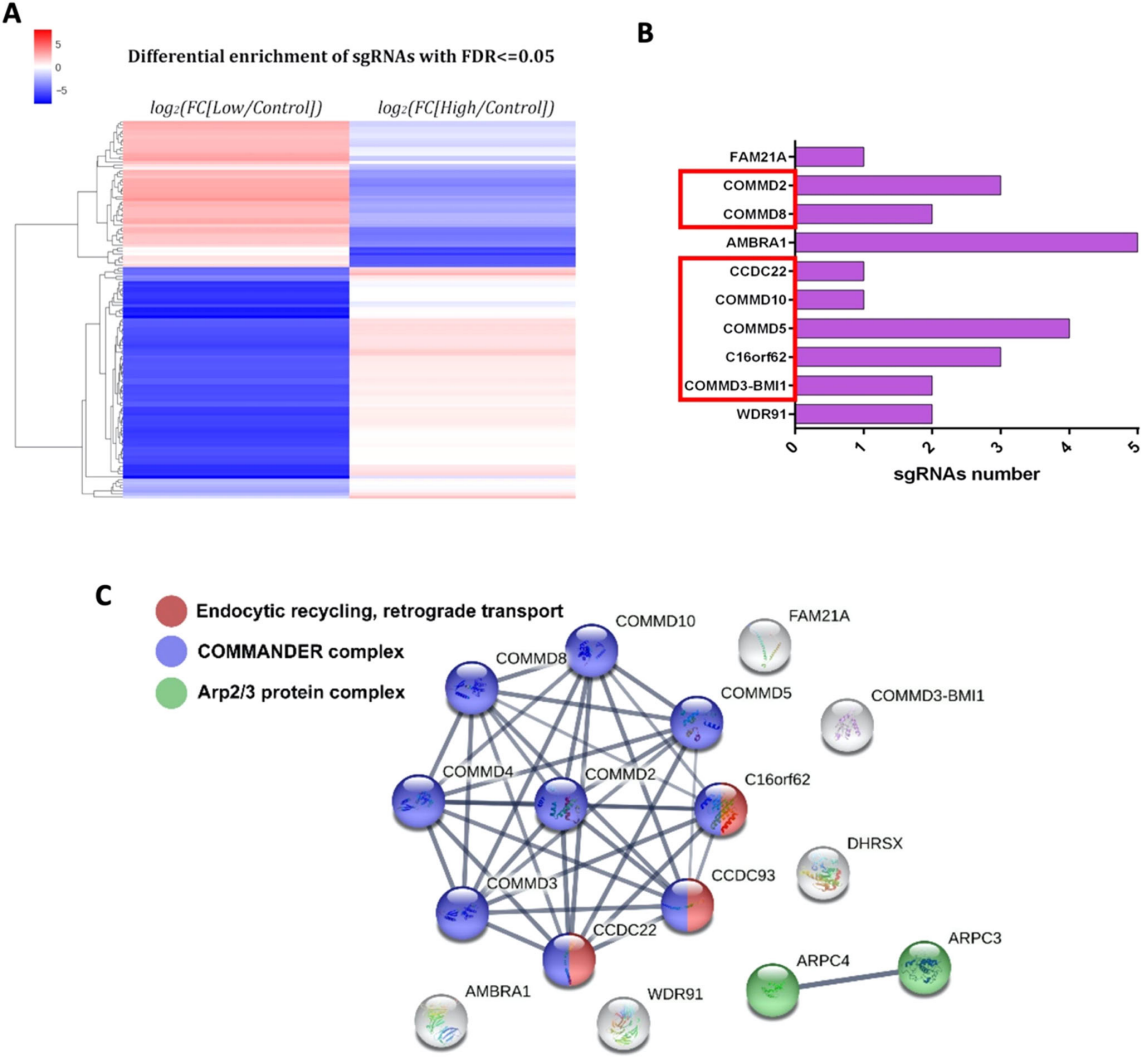
Several components of the COMMANDER complex are included in our screen hit list. (A) Comparison of the enrichment of sgRNAs that change significantly in either of the two datasets, low/control and high/control with each other by using a colour scale in a heatmap. (B) Number of different sgRNAs unveiled in the screen corresponding to the indicated gene among those significantly altered in both set comparisons. (C) STRING ontology analysis of the genes whose sgRNAs are significantly overrepresented in low/control is depicted.

Conversely, the low/control dataset revealed 31 different enriched sgRNAs targeting 16 distinct genes (Figure 2A). Notably, upon comparing this set of enriched genes in the low/control population, an inverse sgRNA enrichment pattern emerged in the high/control dataset, with most of these genes being under‐represented (Figure 3A). Vice versa, the sgRNAs significantly depleted (FC ≤ −1.5) in the High/Control population corresponded to sgRNAs significantly enriched in the low/control set. We thus narrowed our list to those genes that reached significance in both low/control and high/control datasets of our screen, which included COMMD2, COMMD3, COMMD5, COMMD8, COMMD10, AMBRA1, FAM21A, CCDC22, WDR91 and C16orf62 (also known as VPS35L) (Figure 3B). Eight of these genes were represented by multiple sgRNAs in our screen (Figure 3B), further reinforcing the robustness of our analysis.

A large proportion of the hits formed a large cluster in the STRING database, encompassing the COMMANDER complex composed of COMMD2, COMMD3, COMMD4, COMMD5, COMMD8, COMMD10, CCDC22, CCDC93 and C16orf62 (VPS35L) (Figure 3B,C). The COMMANDER complex comprises two multimeric subcomplexes—the CCC complex and the Retriever complex (Singla et al. 2019). Due to functional and structural similarities between the Retriever and the Retromer recycling complex (McNally and Cullen 2018), the COMMANDER complex has been proposed to function primarily in retrograde trafficking pathways from endosomal compartments, although its overall structure is considerably more complex than that of the Retromer complex (Gershlick and Lucas 2017; Singla et al. 2019).

Other prominent hits identified in our genome‐wide assay included the Rab7 effector WDR91 and the WASH complex subunit FAM21A, both of which are also related to recycling activities (Liu et al. 2022; McNally et al. 2017). Additionally, our screen also unveiled the role of the Autophagy and Beclin 1 Regulator 1 (AMBRA1), a key regulator of autophagy and cell signalling, associated with the maintenance of cancer stem cells (Nazio et al. 2021; Cianfanelli et al. 2015) in EV uptake.

Gene set enrichment analyses (GSEA) were conducted on the low/control dataset (Figure S4), which revealed significant enrichment in pathways related to actin nucleation, or intracellular transport routes (early endosome‐to‐Golgi transport, calcium‐regulated exocytosis or oligopeptide transport). Other pathways with positive enrichment included apoptosis, autophagy, mitophagy, regulatory T cell differentiation, insulin signalling or copper metabolism. In these pathways, other hits emerged that had a low score in our previous analyses, such as components of the ARP2/3 complex, Rab14, Rab3GAP1 or genes involved in drug resistance, such as the transporters ABCC1 and ABCB9 (Table S2). Interestingly, although COMMD proteins are known regulators of copper metabolism (Singla et al. 2021), these genes were not annotated under this biological process in the GSEA database. This suggests that the annotation of the COMMANDER complex remains incomplete, potentially limiting the interpretability of these analyses.

**FIGURE 4:**
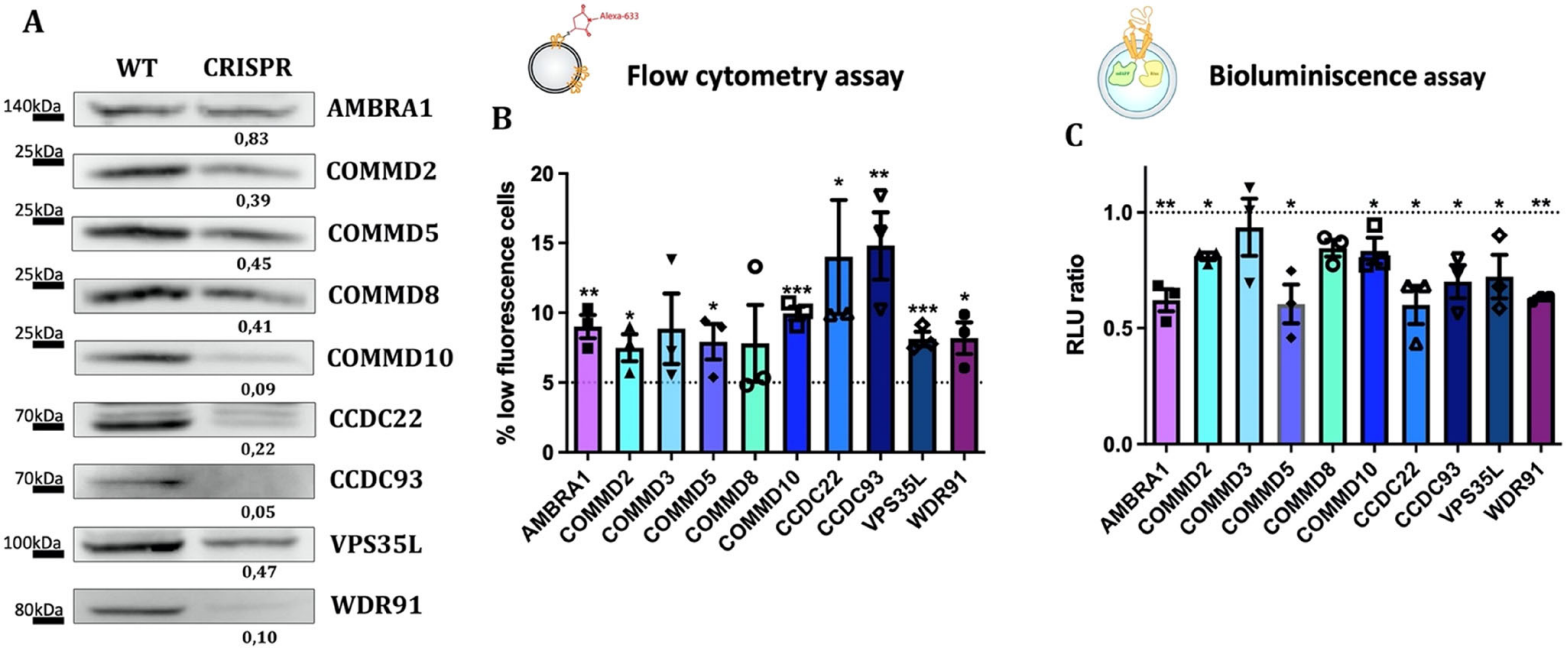
Validations of candidate genes by EV uptake assays. Different CRISPR lines were established in K562 by transduction with the doxycycline‐inducible CRISPR plasmid, TLCV2, carrying the sgRNAs for the different candidate genes selected by the GWC analysis. (A) Expression levels of each protein in its corresponding cell line are shown by Western blot. The densitometry of the bands normalized with respect to the WT line is indicated at the bottom of each image. Their ability to uptake EVs derived from SKMEL‐147 cell line and isolated by SEC was analyzed by flow cytometry using Alexa 633 C5 Maleimide‐labelled EVs (B) or by EnduRen luminescence assay with EVs derived from SKMEL‐147 cells stably expressing the mEMERALD‐CD9‐Rluc construct (C). Horizontal dotted lines depict the levels of WT cells. Data are represented as means ± SEM. *n* = 3. *p ≤ 0.05, **p ≤ 0.01, ***p ≤ 0.001 in one way ANOVA.

For hit validation, we transduced the parental K562 used for the screen with lentiviral vectors carrying the TLCV2 CRISPR/Cas9 plasmid with sgRNAs selected from the hits identified in our analysis. The reduction in protein expression was confirmed by Western blot. Although the resulting populations were not uniformly knockout (Figure 4A), we were able to validate the role of most of these genes in EV uptake using two orthogonal assays. Flow cytometry‐based uptake assays using A633 C5‐maleimide‐labelled EVs showed a significantly higher prevalence of KO cells in the low percentile fluorescence gate (established by gating the 5% low fluorescence cells in the WT cell sample, as done in the initial screen) (Figure 4B). Bioluminescence EV uptake assays, using mEmerald‐CD9‐Rluc EVs (Figure 4C), confirmed these results. Only two of the COMMD molecules (COMMD3 and COMMD8) did not render significant differences, probably reflecting that gene deletion was incomplete and we were analyzing heterogeneous cell populations.

### 3.3 The COMMANDER Complex Is Involved in the Early Phases of EV Uptake by Regulating Adhesion Receptor Expression at the Plasma Membrane

**FIGURE 5:**
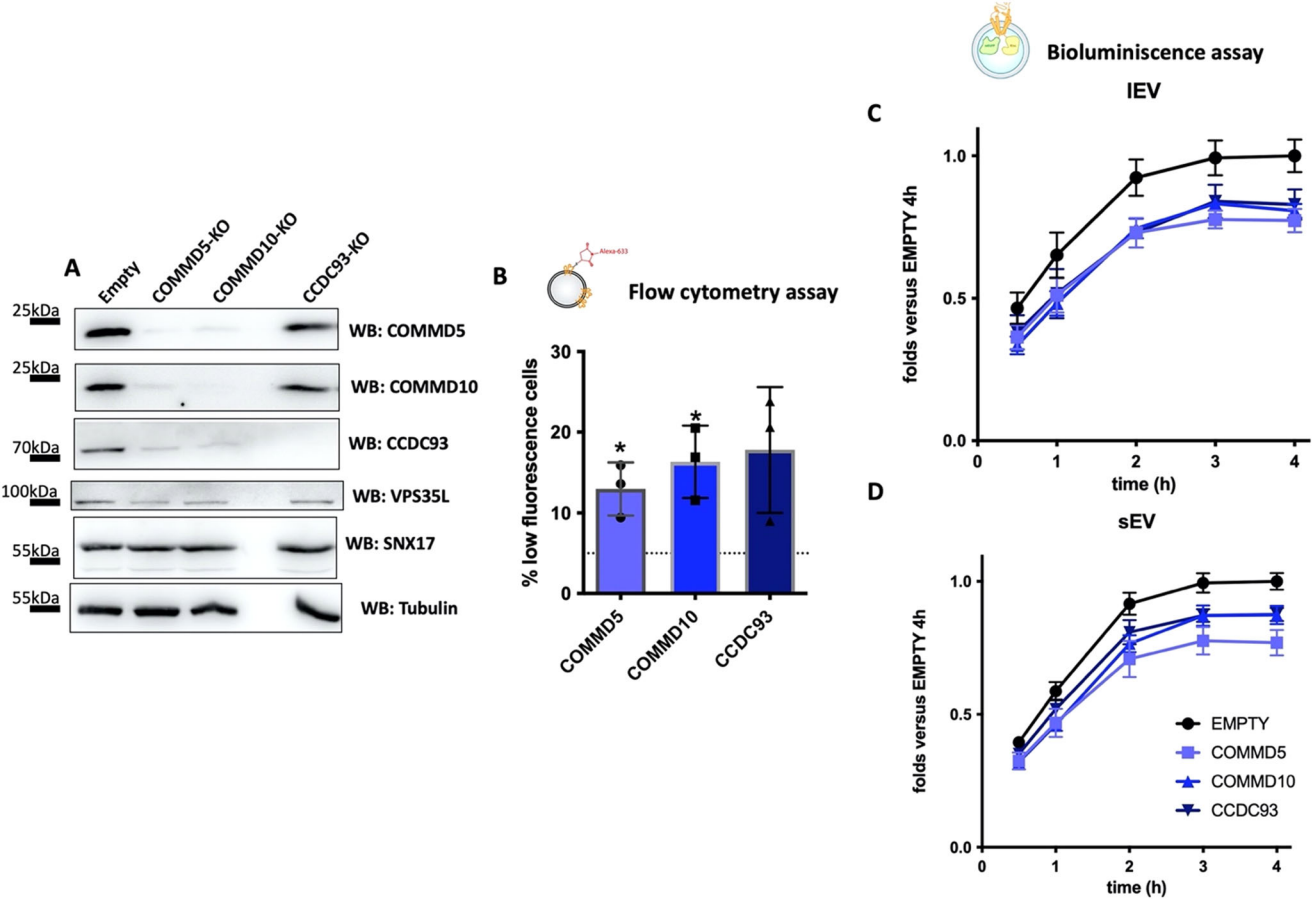
The COMMANDER complex is involved in EV uptake in different cell types and with EVs from different origins. Different CRISPR lines were established in HeLa cells by transduction with the Doxycycline‐inducible CRISPR plasmid, TLCV2, carrying the sgRNAs for the candidate genes of the COMMANDER complex. (A) Expression levels of each protein in its corresponding cell line are shown by western blot. (B) The ability of gene‐deficient cells (deleted gene shown on the *X* axis) to uptake EVs derived from SUM159 cell line measured by flow cytometry using Alexa 633 C5 Maleimide‐labelled EVs, as means ± SEM. *n* = 3. Horizontal dotted line depicts the levels of EMPTY cells *p ≤ 0.05 in one‐way ANOVA. (C) EV Uptake EnduRen luminescence assay with sEVs derived from SUM159 transfected with Renilla‐CD63 ± SEM of 10 different experiments ***p ≤ 0.001 for COMMD5 versus EMPTY, *p ≤ 0.05 for COMMD10 and CDC93 versus empty in a 2way ANOVA or (D) lEV derived from SUM159 transfected with Renilla‐CD9 plotted as means ± SEM of 8 different experiments **p ≤ 0.01 for COMMD5 and COMMD10 versus EMPTY for lEV, *p ≤ 0.05 for CDC93 versus empty in a 2way ANOVA.

Since many of our hits were components of the COMMANDER complex, we decided to focus our attention on the involvement of this complex in EV uptake. To assess the generality of its role, we generated CRISPR‐KO cells in a HeLa cell model of two COMMD members (5 and 10) as well as of CCDC93 (Figure 5A). As previously described for COMMD1 (Bartuzi et al. 2016), gene deletion of COMMD5 or COMMD10 impacted the expression of other COMMANDER members, including other COMMDs, CCDC93 and VPS35L. In contrast, deletion of CCDC93 did not affect the expression of other COMMANDER components (Figure 5A). Using this set of KO cell lines, we quantified EV uptake via the two methods: flow cytometry after maleimide staining or bioluminescence assays. The results confirmed the involvement of the COMMANDER complex in EV uptake, also in this HeLa cell model. Moreover, the effect was consistent when using EVs derived from the melanoma cell line SKMEL‐147 (Figure 5B) or either large or small vesicles isolated from conditioned media of the breast cancer cell line SUM159 (Figure 5C,D).

**FIGURE 6:**
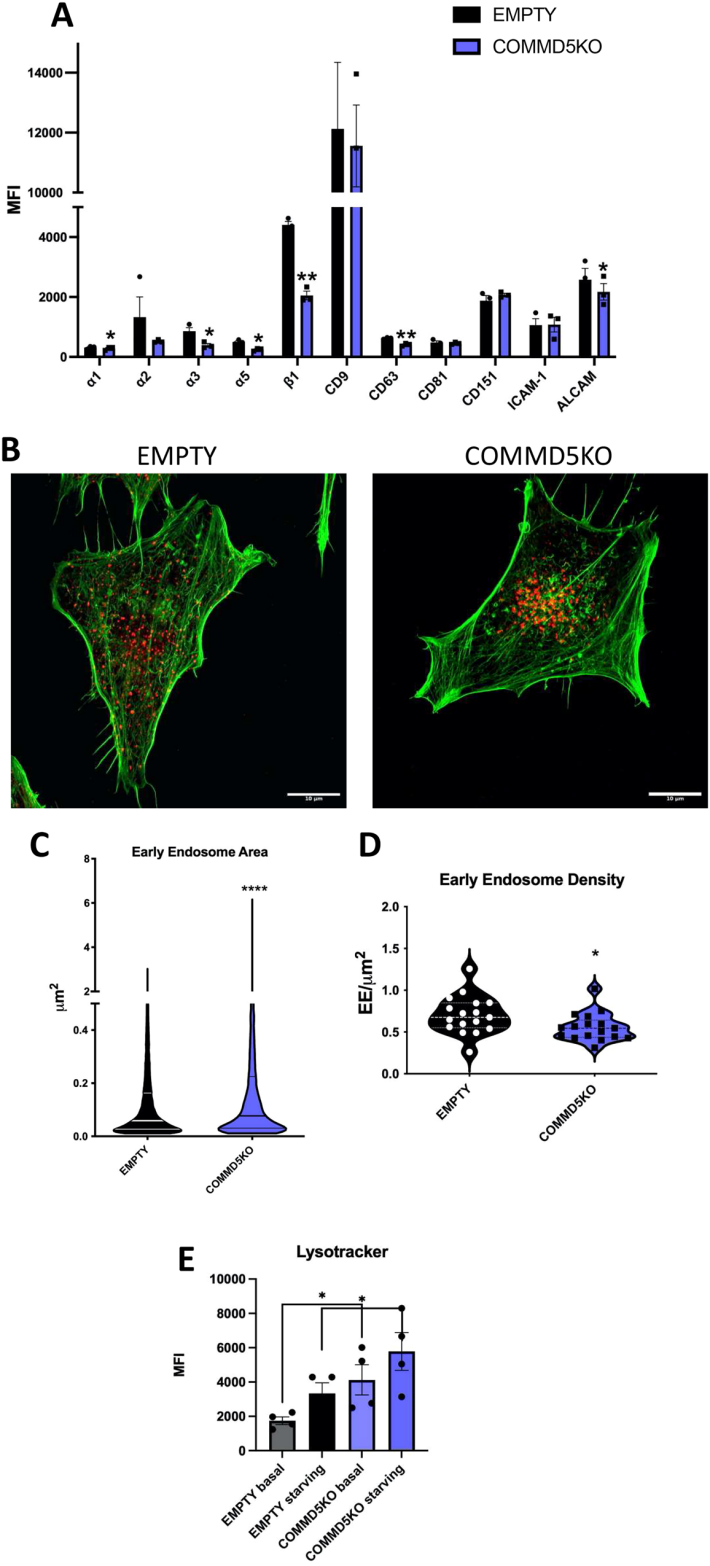
The COMMANDER complex regulates adhesion receptor expression at the plasma membrane and early endosome distribution. (A) Surface expression levels of different adhesion receptors were analyzed by flow cytometry in HeLa cells KO for COMMD5 or their counterparts carrying the vector without sgRNA (EMPTY). Data are shown as means of MFI ± SEM. *n* = 3. *p ≤ 0.05, **p ≤ 0.01 in a paired Student *t*‐test. (B) Confocal microscopy analysis of HeLa cells KO for COMMD5 or EMPTY counterparts stained for early endosomes marker EEA1 (red) and F‐actin (green). Maximal projections of confocal stacks are shown. Bars = 10 µm. The size of individual endosomes (C) and their density per cell (D) were quantified using ImageJ thresholding method. *p ≤ 0.05, ****p ≤ 0.0001 in an unpaired two‐tailed Student *t*‐test. (E) Flow cytometry analyses of acidic compartments using LysoTracker in HeLa EMPTY or COMMD5 KO cells, cultured under standard conditions or starved for 24 h without FBS. The mean fluorescence intensity (MFI) is shown as mean ± SEM. *p ≤ 0.05 in a paired Student *t*‐test.

Both COMMD5 and COMMD10 knockouts had a broad impact on the expression of the entire COMMANDER complex, suggesting they may function as global COMMANDER KOs. Therefore, to investigate the mechanism by which the COMMANDER complex regulates EV uptake, we chose to further characterize the COMMD5 KO cell line in detail. The COMMANDER complex has been involved in the regulation of the recycling and surface expression of several adhesion receptors, including α5β1 integrin (Mcnally et al. 2017). This integrin was recently implicated in the binding and uptake of cancer‐derived exosomes (Cardeñes et al. 2021; De Jong et al. 2020). Our analyses by flow cytometry confirmed reduced plasma membrane expression of α5β1 but also other integrin heterodimers (α2β1, α3β1) as well as ALCAM in COMMD5 KO HeLa cells (Figure 6A). The levels of surface expression of other adhesion receptors, such as ICAM‐1, were not altered. Among tetraspanins, no differences were observed in the expression at the plasma membrane of CD9, CD81 or CD151. However, a reduced expression of CD63 was detected, suggesting a lower exocytic and recycling rate in COMMD5 KO cells.

COMMD5 has previously been shown to mediate endosomal trafficking by connecting endosomes with both actin and tubulin cytoskeletons (Campion et al. 2018). Consistent with this, our COMMD5 KO HeLa cells exhibited a reduced number of early endosomes of a bigger size compared to HeLa cells transduced with an empty vector (Figure 6B,C,D). Early endosomes also showed a tendency to cluster in the perinuclear region in COMMD5KO cells. Moreover, an increase in acidic endosomal compartments was observed in COMMD5 KO cells, as indicated by lysotracker staining (Figure 6E). These findings further support the notion that the COMMANDER complex plays a critical role in early endosome biogenesis and maturation, which may impact EV uptake in acceptor cells.

**FIGURE 7:**
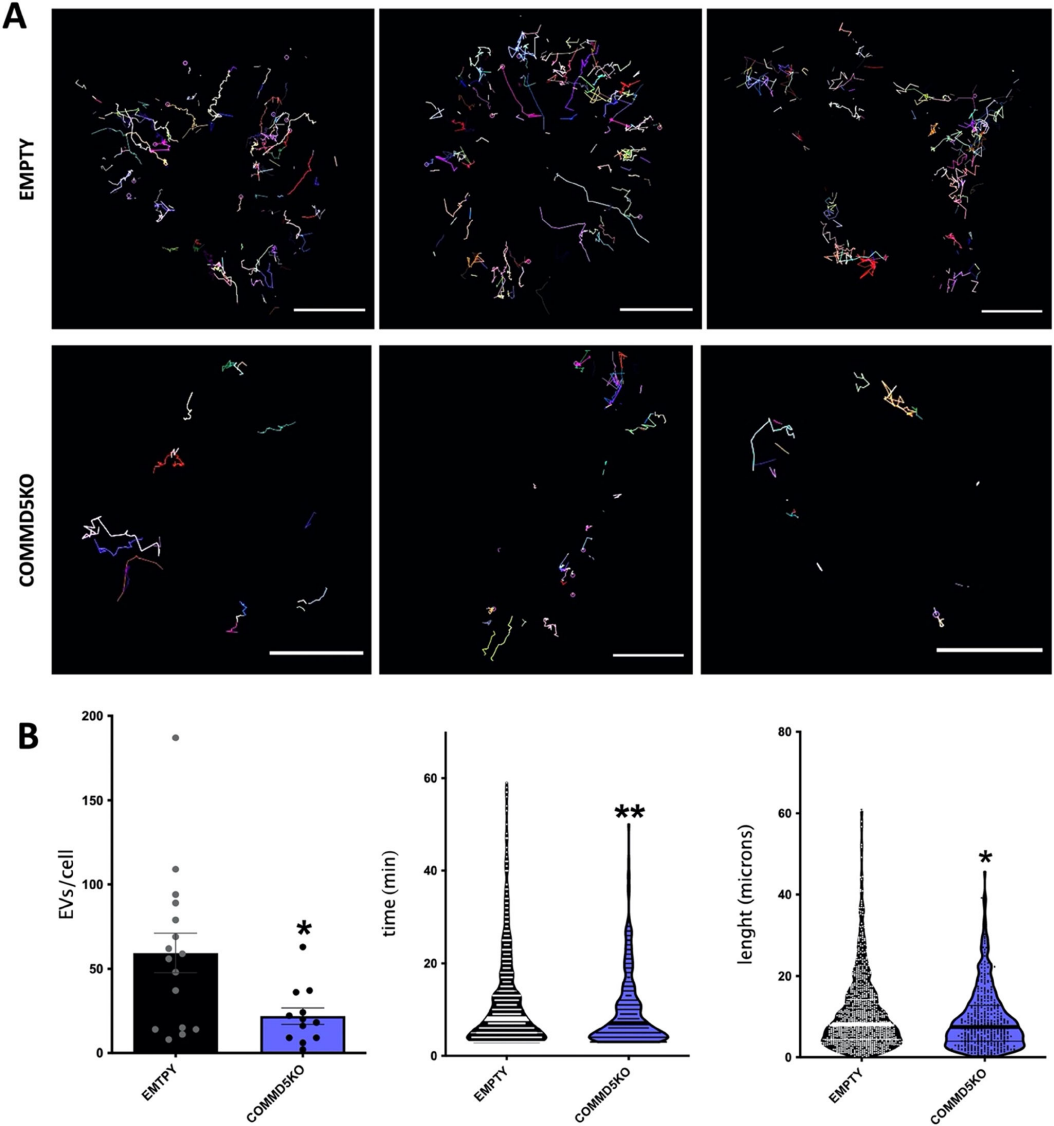
Kinetic analyses of EV uptake by confocal videomicroscopy. (A) HeLa EMPTY vector controls or COMMD5 KO cells were transiently transfected with CD63‐Cherry to visualize endosomal structures. EVs derived from melanoma‐conditioned medium were dual‐labelled: maleimide‐C5 for the surface proteins and CFSE for the lumen. Tracking of all maleimide^+^/CFSE^+^ structures upon contact with the cell was performed for 100 min. Bars = 20µm. (B) Quantitation of the number of tracks/cell (left plot) and the duration (middle plot), and length of the tracks (plot on right). Data correspond to a minimum of 5 cells of each condition from three independent experiments. *p ≤0.05, **p ≤ 0.01 in an unpaired two‐tailed Student *t*‐test.

To gain deeper insight into these processes, we performed time‐lapse videomicroscopy to track the uptake and fate of EVs in COMMD5 KO HeLa cells and their EMPTY vector controls. Both cell lines were transiently transfected with CD63‐Cherry to visualize endosomal structures (magenta in Figure 7 and Supplementary Videos). EVs derived from melanoma‐conditioned medium were dual‐labelled: maleimide‐C5 for the surface proteins (red channel) and CFSE for the lumen (green channel). Double positive (maleimide/CFSE) EVs were tracked over time following contact with HeLa cells (Figure 7A). As expected, COMMD5 KO cells showed reduced interactions with labelled EVs (Figure 7A,B). In both cells, EVs moved towards the cell centre after initial binding at the cell periphery. However, COMMD5 KO cells exhibited significantly shorter EV tracks compared to controls (Figure 7B).

In both samples, the CFSE luminal signal faded more rapidly than maleimide fluorescence, which largely persisted and colocalized with CD63‐positive structures at late time points (see Supplementary Videos). The rapid loss of the CFSE signal suggests EV fusion with the endosomal membrane and subsequent cargo dispersion into the cytosol, though photobleaching cannot be ruled out. Nevertheless, bleaching should occur at comparable rates in both cell lines, as the EVs were from the same batch and samples were simultaneously imaged under identical conditions. Importantly, the shorter duration of double‐positive EV tracks in COMMD5 KO cells supports the conclusion that COMMD5 may also play a secondary role in EV processing after uptake.

**FIGURE 8:**
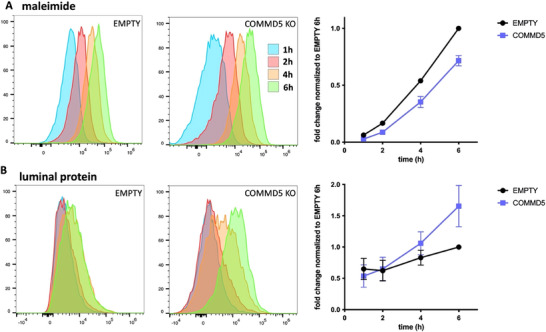
The COMMANDER complex impacts differently on membrane and luminal EV cargo. Kinetic assays of EV uptake by using maleimide labelling of surface proteins (A), and exoglow probe for luminal proteins (B) using Hela cells transduced with an empty vector or with COMMD5 sgRNA guide. Histograms of the kinetic flow cytometry analysis show the overlay of the 1 h point (blue histograms), 2 h (red histograms), 4 h (orange histograms) and 6 h (green histograms). Data are represented as means ± SEM. *n* = 3.

To further analyse the role of the COMMANDER complex in EV cargo delivery or recycling, we conducted quantitative kinetic analysis using EV samples dually labelled with both maleimide (Figure 8A) and a commercial fluorescent probe that stains EV luminal proteins (Figure 8B). Our analyses revealed differences in EV uptake already at the earliest time points for both labelling, further confirming the role of the COMMANDER complex in the initial stages of EV endocytosis. However, while maleimide fluorescence increased more slowly and linearly in COMMD5 KO than in WT cells over time, resulting in a progressively widening difference between the two, the fluorescence from the luminal protein probe showed a different behaviour over time, strikingly recovering at late time points in COMMD5 KO cells and even surpassing the fluorescence levels observed in EMPTY controls. These findings suggest that both types of cargoes (transmembrane and luminal) segregate at some point and undergo differential fates, suggesting that the COMMANDER complex may play additional roles after endocytosis.

## 4 Discussion

CRISPR/Cas9 genetic screens stand as cutting‐edge approaches, offering in a systematic and unbiased fashion a comprehensive functional screening of the entire genome or a designated set of genes. By using CRISPR‐knockout technology, a library of cells harbouring single gene deletions is established. To circumvent potential artefacts stemming from mis‐targeting or unintended gene disruptions, the library incorporates 10 distinct sgRNAs for each gene (Morgens et al. 2017; Lu et al. 2018; Lu et al. 2021). Although CRISPR‐mediated silencing operates at the genomic level, its impacts typically manifest at the translational level. This occurs as the resulting messenger RNAs fail to generate the corresponding proteins due to alterations in the reading frame or the emergence of premature stop codons (Ran et al. 2013; Tuladhar et al. 2019). In addition to gene‐specific targets, the library includes control guides, including those devoid of target sequences anywhere in the genome, as well as guides targeting non‐coding regions such as intronic regions.

In our study, we devised a flow cytometry assay for monitoring EV uptake, enabling sorting of populations exhibiting both higher and lower uptake rates. We utilized maleimide staining, which offers numerous advantages over conventional stains for EV labelling. Maleimide is a small molecule that at neutral pH (6.5–7.5) selectively and covalently binds to thiol/sulfhydryl (‐SH) residues present exclusively on cysteines within proteins. Its non‐hydrophobic nature prevents aggregate formation and diffusion between lipid membranes, minimizing false positive events in EV uptake assays (Toribio and Yáñez‐Mó 2023). Once optimized, maleimide staining provided a wide dynamic range ideal for selecting populations at the extremes of the Gaussian bell curve (Figure S3). The GWC library was established in the K562 leukaemia cell line, chosen as the target due to its ease of handling for flow cytometry and the absence of phagocytic activity, which allows us to focus specifically on more general mechanisms of EV uptake. While the surface adhesion molecule profile of this cell line is relatively limited, K562 cells express α5β1 integrin, a molecule identified as a crucial determinant for EV uptake (Clares‐Pedrero et al. 2024).

Sequencing results of both sorted populations yielded highly consistent outcomes, with the sorted populations exhibiting contrasting sgRNA profiles (Figures 2 and 3). This suggests that the observed phenotypic disparities in our assay led to genuine segregation of distinct single‐gene KO cells, with enrichment in one population and depletion in the other. Particularly relevant were the genes enriched in the low population and depleted in the high population, many of which were targeted by multiple sgRNAs (Figure 3B), underscoring the robustness of our findings.

Prominent hits identified in our genome‐wide assay were the Rab7 effector WDR91 and the WASH complex subunit FAM21A, both of which are also related to recycling activities (McNally and Cullen 2018; McNally et al. 2017), and AMBRA1, a key regulator of autophagy (Nazio et al. 2021; Cianfanelli et al. 2015). GSEA analyses unveiled other hits with lower scores related to actin polymerization, endosomal trafficking or exocytosis.

Strikingly, many of our hits were components of the COMMANDER complex, including COMMD2, COMMD3, COMMD5, COMMD8, COMMD10, CCDC22, CCDC93 and VPS35L (c16orf62). The COMMANDER complex comprises two multimeric subcomplexes—the CCC complex and the Retriever complex (Singla et al. 2019). The CCC complex has been recently characterized through structural studies to form a heterodecamer with ten COMMD proteins arranged in a circular configuration (Healy et al. 2023; Laulumaa et al. 2024; Boesch et al. 2024), while the Retriever complex consists of VPS29, DSCR3 (VPS26C), and the complex's scaffolding protein, VPS35L (c16orf62), the latter two being the ones that interact with SNX17 for cargo recognition (Butkovič et al. 2024) for recycling. Our western blot data is consistent with this overall structure since gene deletion of COMMD5 resulted in an impaired expression of COMMD10 and vice versa. Cryo‐electron microscopy data supporting this model also reveal that the interaction of CCDC22 and CCDC93 is vital for stabilizing the heterodecamer formation (Healy et al. 2023; Laulumaa et al. 2024; Boesch et al. 2024). Again, our western blot data also support a stabilizing role for CCDC93 (Figure 5A). Notably, the COMMANDER complex exhibits a high level of conservation, with interactions among orthologs of CCDC22, CCDC93, and VPS35L observed even in unicellular protozoa like *Dictyostellium* (Liebeskind et al. 2019; Maine and Burstein 2007) and several COMMD mouse KO models showing embryonic lethality (Van De Sluis et al. 2007; Li et al. 2015).

At the functional level, much data support a role of the COMMANDER complex in endosomal membrane recycling, similar to the retromer complex, despite its structure being considerably more complex. COMMD1 orchestrates the retrograde transport of ATP7A and ATP7B, essential for copper transport, so that a deficiency in COMMD1 leads to copper toxicity syndromes (Vonk et al. 2011). COMMD1, COMMD6 and COMMD9 regulate the recycling of LDLR receptors by associating with the WASH complex. Their deficiency is associated with hypercholesterolaemia and an elevated risk of atherosclerosis (Bartuzi et al. 2016; Fedoseienko et al. 2018). COMMD9 governs the membrane recycling of Notch2, preventing its degradation and thereby influencing conditions related to the WAGR syndrome (Li et al. 2015). COMMD5 oversees the recycling of ligand‐activated EGFR and regulates endosomal trafficking through interactions with actin, tubulin and Rab5 (Campion et al. 2018). COMMD2, COMMD3, COMMD4 and CCDC22 have been implicated in the recycling of SARS‐CoV‐2 viral particles in CRISPR screening assays (Daniloski et al. 2021). Finally, a recent study highlights the role of COMMANDER in the maintenance of lysosomal homeostasis and regulation of EV release (Minakaki et al. 2025).

Our GWC screen results indicate that the COMMANDER complex plays a relevant role in EV uptake. We demonstrated that this role is conserved across different cell types and EV sources, further supporting the relevance of our findings. A possible mechanism for this effect could be that the absence of the COMMANDER complex resulted in mis‐trafficking of critical cell surface receptors essential for adhesion or engagement with extracellular vesicles (EVs), ultimately hindering their uptake. Indeed, our data revealed that COMMD5 KO cells showed a reduced plasma membrane expression of several integrin heterodimers but also of ALCAM, all of which have been demonstrated to influence EV binding and uptake (Clares‐Pedrero et al. 2024; Cardeñes et al. 2022). Interestingly, while the GWC screening aimed to identify proteins involved in EV uptake, no specific integrin or receptor emerged as a significant hit. This suggests that EV binding to target cells may rely on multiple redundant receptors. Consistently, deletion of COMMANDER, which impacted the expression of several adhesion receptors, produced a detectable effect.

Our screening assay analyzed cell populations based on their fluorescence intensity derived from the uptake of maleimide‐labelled EVs. For this reason, the specific role of the COMMANDER complex—whether in endocytosis, cargo delivery, or the recycling of internalized components—could not be precisely determined. To address this limitation, we performed a series of validation experiments to further characterize the involvement of the complex in each of these processes. In our uptake assay, labelling was limited to integral membrane proteins, as only proteins with available cysteines on their extracellular domains were tagged. Similarly, in the bioluminescence assay, the label was attached to CD9 or CD63, transmembrane EV proteins. Consequently, a reduction in signal within target cells could either result from reduced EV uptake or from impaired recycling of the cargo to the plasma membrane, which would ultimately lead the cargo to a degradative pathway. The COMMANDER complex could also influence the back fusion of the EV membrane with the limiting endolysosomal lipid bilayer to release the luminal cargo. To directly address these possibilities, we conducted a new set of experiments with COMMD5‐KO HeLa cells, comparing the uptake of EVs labelled with maleimide (tagging surface proteins) and fluorescent probes tagging the luminal content. The kinetic analysis revealed that both EV cargo types were similarly affected at early time points, further confirming that the COMMANDER complex is involved in the early phases of EV uptake, likely at the endocytic level. However, at later time points, the behaviours of transmembrane and intraluminal cargo diverged. The transmembrane cargo signal remained diminished throughout the kinetic experiment in the KO cell line, suggesting a cumulative effect of reduced uptake. In contrast, the intraluminal cargo signal in COMMD5 KO cells recovered by the 2 h time point and even exceeded the WT signal at the 6 h time point. These surprising findings suggest that transmembrane and intraluminal cargo become decoupled after endocytosis, supporting the hypothesis of endosomal escape via EV membrane back fusion. In agreement with this model, the duration of tracks from double positive structures was shorter in COMMD5 KO cells. In most of these events, the maleimide fluorescence was maintained, further suggesting a fusion event. Once the luminal cargo accesses the cytosol, it may escape from degradation routes, thus explaining the accumulation in target cells observed in flow cytometry experiments. Nevertheless, several technical limitations of these experiments, such as spatial resolution and the use of indirect labelling of EV cargoes with fluorescent small molecules, which could potentially dissociate from their original EV cargoes upon lysosomal degradation and proteolytic cleavage, are difficult to circumvent. In addition, disruption of COMMANDER has recently been reported to increase EV release (Minakaki et al. 2025), so we cannot rule out the possibility that loss of COMMD5 leads to enhanced re‐exocytosis of endocytosed EVs, although this kind of event was not detected in the video microscopy experiments. Further work will be needed to elucidate the exact role of COMMANDER in EV cargo fate and delivery in target cells.

Extracellular vesicles have emerged as a pivotal concept in cell–cell communication, playing essential roles in biological processes, while also showing promise as potential non‐invasive disease biomarkers and drug delivery vehicles. Understanding the molecular underpinnings of EV uptake is paramount for the clinical applications of EVs, particularly as they progress into ongoing clinical trials. Achieving specific tissue and cell delivery is crucial for enhancing the safety and efficacy of EV‐based therapeutics in real clinical practice. Furthermore, a comprehensive molecular elucidation of the intricacies involved in EV cargo delivery to target cells has the potential to reveal innovative therapeutic targets. This could significantly expand the arsenal of immunotherapy tools to enhance anti‐tumour immune responses, thereby overcoming some of the current limitations in these approaches.

## Author Contributions

**Miguel Palma‐Cobo**: Formal analysis (equal); investigation (equal); validation (equal); writing ‐ original draft (equal). **Victor Toribio**: Data curation (equal); formal analysis (equal); investigation (equal); validation (equal); writing ‐ original draft (equal). **Joaquín Morales**: Investigation (supporting); methodology (supporting). **Soraya López‐Martín**: Investigation (equal); methodology (equal); project administration (equal). **Carlos Enrich**: Resources (equal). **Albert Lu**: Conceptualization (equal); data curation (equal); methodology (equal); resources (equal); supervision (equal); writing ‐ review and editing (equal). **María Yáñez‐Mó**: Conceptualization (equal); data curation (equal); formal analysis (lead); funding acquisition (lead); methodology (equal); project administration (equal); supervision (lead); writing ‐ review and editing (lead).

## Conflicts of Interest

The authors declare no conflicts of interest.

## Supporting information




**Supplementary Video KO1, KO2, KO3**. COMMD5 KO HeLa cells were transiently transfected with CD63‐Cherry to visualize endosomal structures (magenta channel). EVs derived from melanoma‐conditioned medium were dual‐labelled: maleimide‐C5 for the surface proteins (red channel) and CFSE for the lumen (green channel). The maximal projection of the three optical sections acquired is shown. Cells from the videos correspond to the tracks in Figure 7A.

**Supplementary Video WT1, WT2, WT3**. HeLa EMPTY vector control cells were transiently transfected with CD63‐Cherry to visualize endosomal structures (magenta channel). EVs derived from melanoma‐conditioned medium were dual‐labelled: maleimide‐C5 for the surface proteins (red channel) and CFSE for the lumen (green channel). The maximal projection of the three optical sections acquired is shown. Cells from the videos correspond to the tracks in Figure 7A.

**Supplementary Material**: jev270166‐sup‐0003‐SuppMat.docx

## Data Availability

The data that support the findings of this study are available from the corresponding author upon reasonable request.
