# Supplementary material for: Genome‐Wide CRISPR/Cas9 Screening Identifies the COMMANDER Recycling Complex as a Key Player in EV Uptake
Source: J Extracell Vesicles. 2025 Sep 23;14(9):e70166. doi: 10.1002/jev2.70166 (PMC12456102; doi:10.1002/jev2.70166)
Supplement: Supplementary file 3 — Supplementary Material: jev270166‐sup‐0003‐SuppMat.docx [file JEV2-14-e70166-s003.docx]

**Supplementary Table 1:**

**Primers employed in the GWC assay.**

First PCR:

-Forward primer_oMCB1562: 5’AGGCTTGGATTTCTATAACTTCGTATAGCATACATTATAC 3’.

-Reverse primer_oMCB1563: 5’ACATGCATGGCGGTAATACGGTTATC 3’.

Second PCR:

-Forward primer_oMCB1439: P5 Illumina sequence (RED)

5’CAAGCAGAAGACGGCATACGAGATGCACAAAAGGAAACTCACCCT 3’.

-Reverse barcoded CRISPR KO primers: P7 Illumina sequence (RED). One index (GREEN) per sample was used.

Index #1 (used in Low 1) 5’AATGATACGGCGACCACCGAGATCTACACGATCGGAAGAGCACACGTCTGAACTCCAGTCAC GGTAGCCGACTCGGTGCCACTTTTTC 3’.

Index #2 (used in Low 2) 5’AATGATACGGCGACCACCGAGATCTACACGATCGGAAGAGCACACGTCTGAACTCCAGTCAC TAATCGCGACTCGGTGCCACTTTTTC 3’.

Index #3 (used in High 1) 5’AATGATACGGCGACCACCGAGATCTACACGATCGGAAGAGCACACGTCTGAACTCCAGTCAC CCAACACGACTCGGTGCCACTTTTTC 3’.

Index #4 (used in High 2) 5’AATGATACGGCGACCACCGAGATCTACACGATCGGAAGAGCACACGTCTGAACTCCAGTCAC CAGGCGCGACTCGGTGCCACTTTTTC 3’.

Index#5 (used in Unsorted 1) 5’AATGATACGGCGACCACCGAGATCTACACGATCGGAAGAGCACACGTCTGAACTCCAGTCAC CACTCACGACTCGGTGCCACTTTTTC 3’.

Index #6 (used in Unsorted 2) 5’AATGATACGGCGACCACCGAGATCTACACGATCGGAAGAGCACACGTCTGAACTCCAGTCAC CATGGCCGACTCGGTGCCACTTTTTC 3’.

Illumina NGS:

Custom Sequencing primer: 5’GCCACTTTTTCAAGTTGATAACGGACTAGCCTTATTTAAACTTGCTATGCTGTTTCCAGCTTAGCTCTTAAAC 3’.


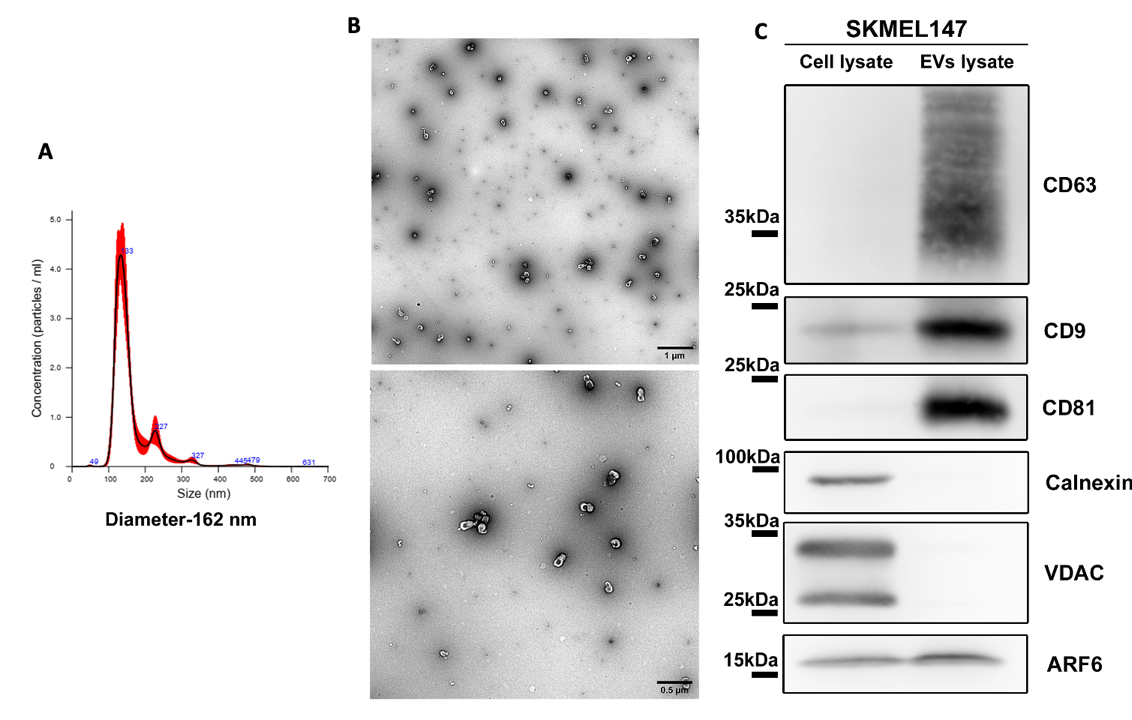


**Supplementary Figure 1. Characterization of EVs derived from the melanoma cell line SKMEL-147.** Total EVs isolated by ultracentrifugation at 100,000g were characterized by **(A)** Nanoparticle tracking analysis (NTA) and **(B)** transmission electron microscopy by negative staining with uranyl salts. Bars=1μm (upper image), 0.5μm (lower image). **(C)** Analysis of EVs samples by immunoblotting with the EVs positive markers CD63, CD9, CD81 and ARF6 and the cell markers Calnexin and VDAC.


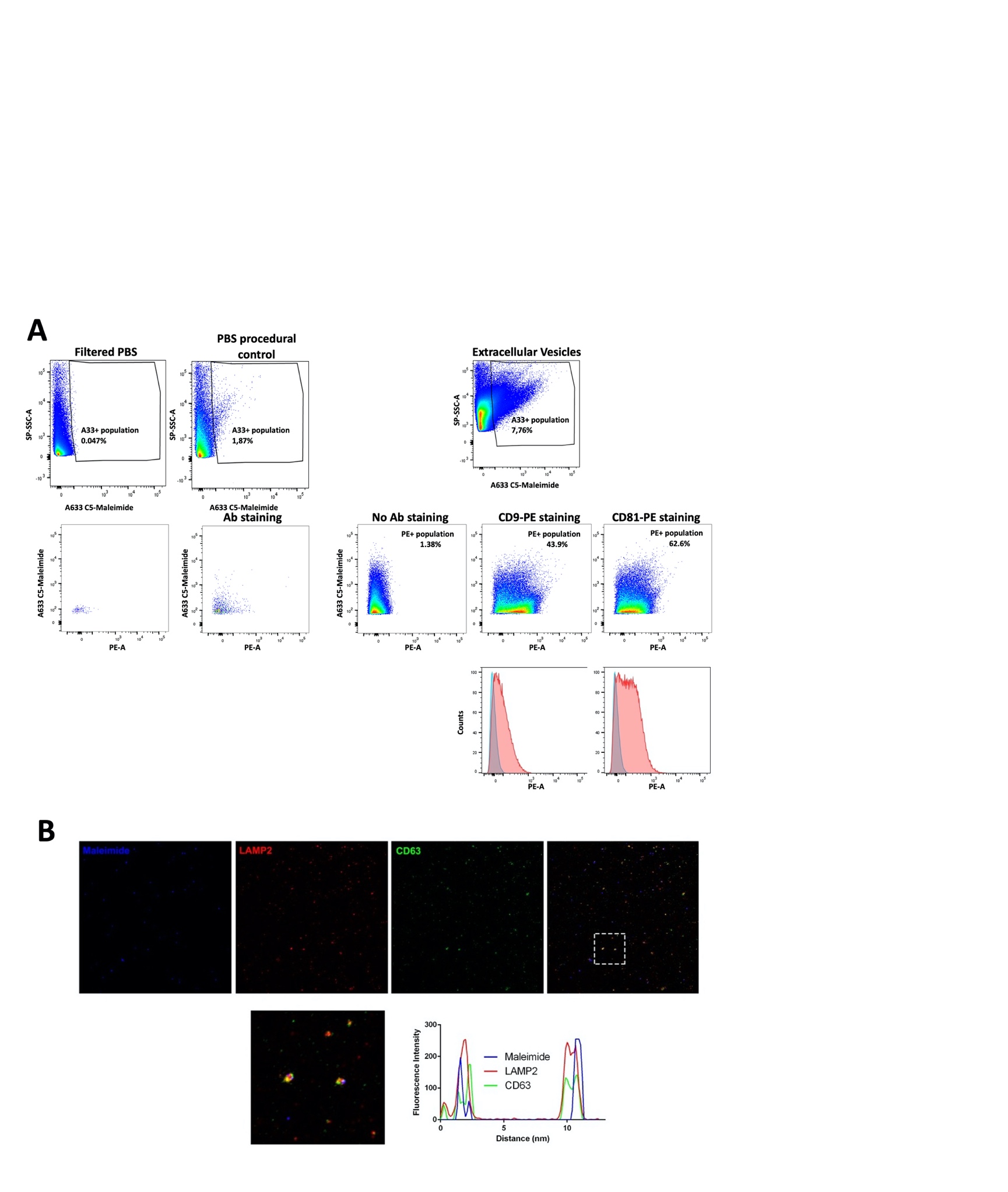


**Supplementary Figure 2.** **Characterization of EV labelling with fluorescent Maleimide. (A)** Flow cytometry analysis of SKMEL-147 derived EVs stained with fluorescent Maleimide is shown. The general population was selected by Alexa633-Maleimide labelling (see gate in top panels) and then analyzed for labelling with α-CD9-PE or α-CD81-PE. A procedural control PBS treated the same as the EVs samples (Maleimide and Ab cocktail staining followed by SEC) was used and to determine the signal background and specificity. Maleimide/PE dot plots for the gated population are shown for all samples (middle panels). In vesicle samples PE signal histogram plots of the overlay of stained samples with noAb signal are also depicted (lower panels). Similar results were obtained upon sample dilution (not shown), ruling out a swarm effect. **(B)** Confocal microscopy analysis of the Alexa633-Maleimide EV labelling (blue), in an immunolabeling with α-LAMP2 (red) and α-CD63 (green). A graph is shown with the fluorescence intensities of the EVs shown in the enlarged image. Although confocal imaging lacks the resolution to distinguish single vesicles from aggregates, it clearly demonstrates the specificity of the maleimide staining, as no maleimide-positive signals were detected as EV-negative dots.

**
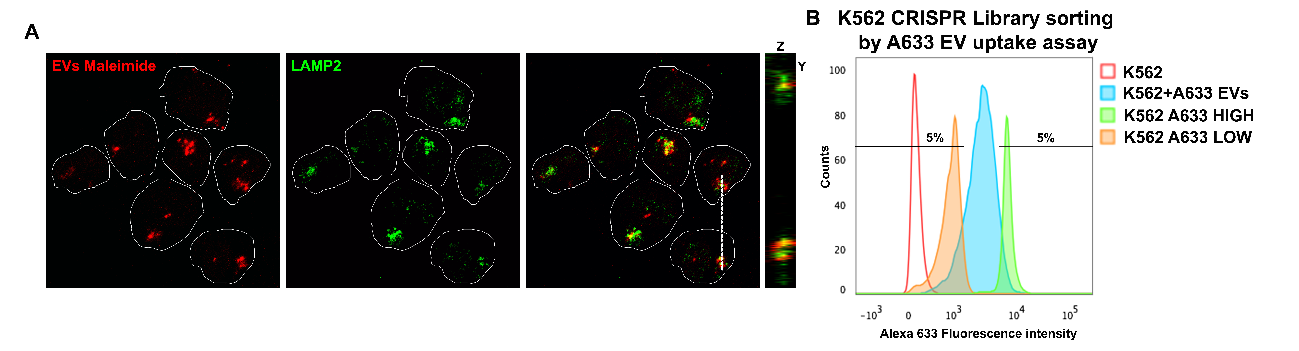
**

**Supplementary Figure 3. Uptake assay with Maleimide-labelled EVs and GWC library screening of populations of interest. (A)** Confocal microscopy images of K562 cells incubated for 2 hours with Alexa633 C5-Maleimide-labelled EVs (red) and then immunostained with α-LAMP2 to view lysosomal compartments. A vertical section is shown on the right. **(B)** Histogram of the flow cytometry sorting assay performed with the CRISPR cell library and Alexa633 C5-Maleimide-labelled EVs. Shown are the untreated control K562 cells (red line on histogram), the total population after being incubated for 2 hours with the labelled EVs (blue background histogram) and the high (High) (green) or low fluorescence (Low) (orange) populations in a post-screening analysis.

**Supplementary Figure 4:** The c5.go.bp.v2025.1.Hs.symbols.gmt gene set collection was used to look for gene ontology in biological processes using 1000 permutations with a threshold of at least 15 genes per gene set. The score for the pre-reanked GSEA was calculated multiplying -log10(FDR) with the log2 fold change of Low VS Control. Since in our library we have 10 sgRNA for each gene and GSEA takes only one entry per gene, the final score was calculated as the mean of all the scores for the same gene.

**Supplementary Table 2:** List of genes with positive core enrichment in the different pathways unveiled in the GSEA analysis. Pre-ranking of the Low VS Control dataset was performed using the mean of the scores for the 10 sgRNA for each gene calculated as -log10(FDR) multiplied by the log2 fold change

**Supplementary Video WT1, WT2, WT3.** HeLa EMPTY vector control cells were transiently transfected with CD63-Cherry to visualize endosomal structures (magenta channel). EVs derived from melanoma-conditioned medium were dual-labeled: maleimide-C5 for the surface proteins (red channel) and CFSE for the lumen (green channel). The maximal projection of the three optical sections acquired is shown. Cells from the videos correspond to the tracks in Figure 7A.

**Supplementary Video KO1, KO2, KO3.** COMMD5 KO HeLa cells were transiently transfected with CD63-Cherry to visualize endosomal structures (magenta channel). EVs derived from melanoma-conditioned medium were dual-labeled: maleimide-C5 for the surface proteins (red channel) and CFSE for the lumen (green channel). The maximal projection of the three optical sections acquired is shown. Cells from the videos correspond to the tracks in Figure 7A.
